# The Role of Rare Earth Lanthanum Oxide in Polymeric Matrix Brake Composites to Replace Copper

**DOI:** 10.3390/polym10091027

**Published:** 2018-09-14

**Authors:** Kaikui Zheng, Chenghui Gao, Fushan He, Youxi Lin

**Affiliations:** 1 School of Mechanical Engineering and Automation, Fuzhou University, Fuzhou 350116, China; kuikui@fzu.edu.cn (K.Z.); hfshan@fzu.edu.cn (F.H.); lyx@fzu.edu.cn (Y.L.); 2 Mechanical and Electrical Engineering Practice Center, Fuzhou University, Fuzhou 350116, China

**Keywords:** rare earth, lanthanum oxide, copper, polymeric, brake composites, tribological properties

## Abstract

The main focus of current research in polymeric matrix brake composites is on searching out a replacement for copper, which has been recently proved to be a hazard to human health and the environment. In this paper, rare earth lanthanum oxide was explored for the replacement of copper in composites. The mechanism of the role of lanthanum oxide in brake composites to replace copper was analyzed. Four series of polymeric matrix brake composites with various amounts of copper (15, 10, 5 and 0 wt %) and rare earth lanthanum oxide (0, 5, 10 and 15 wt %) were developed, in which the copper was gradually replaced by lanthanum oxide in the formula. These series were characterized in terms of physical, thermo-physical and mechanical properties. The results show that lanthanum oxide can be successfully used as a replacement for copper in brake composites. Brake composites with 15 wt % lanthanum oxide that are copper-free are considered optimal, where tribo-properties are considered best. Compared with the addition of copper in brake composites, lanthanum oxide is more conducive to the formation of compacted friction films and transfer films, which is beneficial to the tribological properties of the brake composites. The addition of La_2_O_3_ to the brake composites can cause the reaction between La_2_O_3_ and Al_2_O_3_ to form LaAlO_3_, and the reaction between Al_2_O_3_ and BaSO_4_ can produce Ba_18_Al_12_O_36_ and Al_2_SO_4_ during the friction and wear processes, which can effectively improve the tribological properties of the brake composites at elevated temperature. This research was contributive to the copper-free, metal-free and eco-friendly brake composites.

## 1. Introduction

During braking, the wear debris, which contains several hazardous elements (e.g., copper, lead, zinc, antimony), has been recently proven as a threat to human and aquatic life [1,2,3]. In 2010, California and Washington State pioneered new laws to restrict copper and other heavy metals in brake pads [4]. With the development of the automobile industry, requirements for safety, comfort and environmental friendliness are highly demanded. The development of copper-free, metal-free and eco-friendly brake composites will be a trend in this field. The non-metallization of the polymeric matrix brake composites must be realized by looking for substitute materials of copper.

Copper is an important component in polymeric matrix brake composites and plays a crucial role in the friction properties of the brake composites. The positive effects of copper on brake composites can be summarized in three aspects. First, copper has high thermal conductivity and can effectively conduct away heat from the friction interface, contributing to the good heat resistance of the brake composites [5,6]. Second, copper is often used in the friction material industry as a solid lubricant for high-temperature applications that maintain the stability of friction at elevated temperatures [7,8]. Third, copper is also known for its good ductility, contributing to the formation of friction films, playing an important role in friction and wear properties [9,10,11]. Many scholars worldwide are conducting research to develop brake composites formulations without copper, and some achievements have been reached [12,13,14,15,16,17]. However, most developed copper-free brake composites have weaker friction properties than the copper-containing formulations, and most of them contain one or more types of steel fiber, metal lubricants, metal oxides, etc., which cannot realize polymeric matrix brake composites without metals. Gilardi et al. [18] studied the performance of copper-free brake composites by replacing copper with various types of graphite and reported that different types of graphite and their combined use improved the noise vibration and harshness performance and thermal conductivity of the composites. Aranganathan and Bijwe [19] studied the effect of newly commercially available thermo graphite on the friction properties of copper-free friction materials and concluded that this graphite showed low fade and fluctuations in friction coefficient, but they drastically reduced other friction performance, which were not desirable features. Copper perhaps must be replaced by a combination of ingredients. There has been no single ingredient that can replace copper in brake composites.

Rare earth oxides as inorganic particles are gradually introduced into the preparation of the composites because of their excellent physical and chemical characteristics that improve the properties of many materials. It can be used in composite materials to improve the mechanical properties, interfacial properties, heat resistance, etc. [20,21,22,23]. The oxides are allotropes of hexagonal crystals, which are similar to layered structures with high melting points and low hardness, act as a solid lubricant at high temperature [24]. In our previous works [25,26], the effects of rare earth lanthanum oxide on the mechanical and tribological properties of the brake composites were studied. We found that rare earth lanthanum oxide can significantly improve the mechanical and tribological properties of the brake composites. Based on these findings, this paper studies the mechanism of the role of rare earth lanthanum oxide in brake composites by replacing copper with rare earth oxides. Hence, four series of polymeric matrix brake composites with varying amounts of copper and rare earth lanthanum oxide were designed and developed by reducing the content of copper (15, 10, 5 and 0 wt %) while increasing the corresponding content of lanthanum oxide (0, 5, 10 and 15 wt %) to replace copper.

## 2. Materials and Methods

### 2.1. Formulation and Designation of Composites

**Figure 1:**
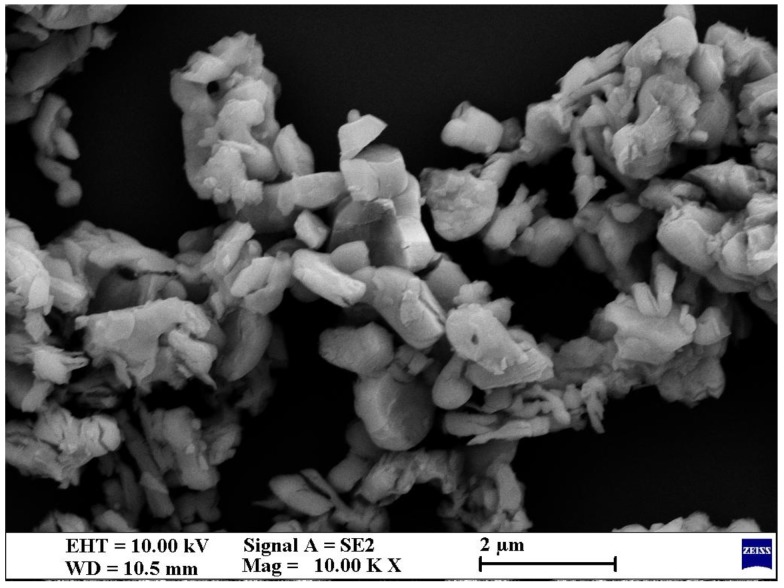
Scanning electron microscope (SEM) micrograph of selected La_2_O_3_.

**Figure 2:**
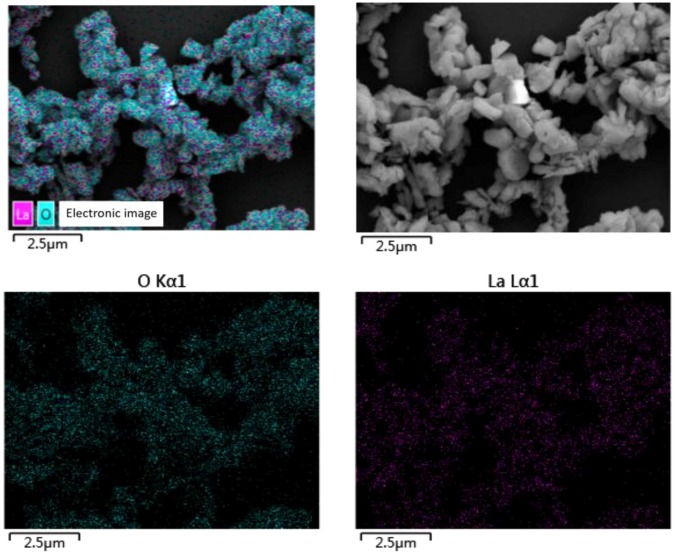
Energy dispersive spectrometry (EDS) elemental mapping images of selected La_2_O_3_.

Rare earth lanthanum oxide (La_2_O_3_) was supplied by Foshan blue pigment New Material Co., Ltd. (Foshan, China). Table 1 outlines the details of La_2_O_3_, which were provided by the supplier. La_2_O_3_ powder was examined by scanning electron microscopy (SEM) (Model SUPRA 55, Carl Zeiss AG, Jena, Germany) and energy dispersive spectrometry (EDS) (Model X-Max50, Oxford Instruments Co., Ltd., Oxford, UK) to study the details of the shape, size, and elements, as shown in Figure 1 and Figure 2. Ceramic fibers was supplied by Zhoukou Qifeng Mineral Fibers Co., Ltd. (Zhoukou, China). Table 2 outlines its details, which were provided by the supplier. Cashew nut-shell-liquid-modified phenolic resin was supplied by Sumitomo (Tokyo, Japan). Table 3 outlines its details, which were provided by the supplier. Amongst eight ingredients of the brake composites, except La_2_O_3_, ceramic fibers and phenolic resin, all fillers such as copper (Cu), graphite, alumina, barite and nitrile-butadiene rubber (NBR) powder were procured from a local supplier (Fuzhou Taijiang Pinjie Experimental Instrument Co., Ltd., Fuzhou, China). Table 4 outlines the details of Cu, which were provided by the supplier.

Brake composites containing eight ingredients were formed by keeping 85 wt % fixed (comprising fibres, additives and various fillers) as a parent composition. The balance 15 wt % was adjusted by varying the wt % of Cu as 15, 10, 5 and 0 wt % and compensated with La_2_O_3_ (the replacement of Cu). The prepared composites were designated as C_15_L_0_, C_10_L_5_, C_5_L_10_ and C_0_L_15_. The full design of the series is shown in Table 5.

### 2.2. Fabrication of Composites

The ingredients were mixed in a plough type shear mixer (Model JF810S, Jilin Electrical and Mechanical Equipment Co., Ltd., Changchun, China) to ensure macroscopic homogeneity. The mixing was performed for 15 min. Then, the mixture was placed in a four-column hydraulic machine (Model Y32-63, Ruian Huada Machinery Co., Ltd., Ruian, China). The mold cavity was filled with approximately 60 g of the mixture and heat-cured in a compression molding machine under a pressure of 10 MPa for 10 min, at a curing temperature of 160 °C. Five intermittent breathings were provided to allow the volatiles to be expelled during the initiation of curing. Then, the brake composites were post-cured in an oven (Model JF980S, Jilin Electrical and Mechanical Equipment Co., Ltd., Changchun, China) at 160 °C for 12 h. The post-cured brake composites were surface-grinded, polished, and used for further characterization.

### 2.3. Characterization of Brake Composites

#### 2.3.1. Characterization of Physical, Mechanical, and Thermo-Physical Properties

The developed brake composites were characterized for physical (density and porosity), mechanical (hardness and impact strength) and thermo-physical (thermal conductivity and thermal resistance) properties. The density of the brake composites was calculated based on Archimedes principle. The porosity was measured using the Japanese Industrial standard JIS D 4418-1996 [27], and the porosity, *p*, can be expressed as

(1) $$p=\frac{m_{1}-m_{0}}{\rho }\frac{1}{V}\times 100\%$$

where $m_{0}$ is the initial mass of the sample, $m_{1}$ is the mass of the sample after oil absorption, *ρ* is the density of the sample, and *V* is the volume of the sample. The hardness was measured using an electric plastic Rockwell hardness tester (Model XHRD-150, Laizhou Huayi Test Instrument Co., Ltd., Laizhou, China), according to the ISO 2039-2:1987 standard [28]. The impact strength of the brake composites was measured using a simply-supported beam pendulum impact tester (Model XJJ-5, Jinan Fangyuan Test Instrument Co., Ltd., Jinan, China), according to the ISO 179-1:2010 standard [29]. The impact strength, $\alpha_{k}$, can be calculated by

(2) $$\alpha_{k}=\frac{A}{b\cdot d}\times 10^{3}$$

where *A* is the energy required for breaking the sample, *b* is the width of the sample, and *d* is the thickness of the sample. The tensile strength and elastic modulus of the composites were measured using a microcomputer-controlled electronic universal testing machine (Model CMT-5105, New Sice Materials Testing Co., Ltd., Shenzhen, China), according to the ISO 527-4 standard [30]. Thermo-physical properties such as thermal conductivity and thermal resistance of the brake composites were measured using a thermal conductivity tester (Model DRL-II, Xiangtan Xiangyi Instrument Co., Ltd., Xiangtan, China), according to the ASTM D5470-2006 standard [31]. Each case was measured five times, after which the results were averaged.

#### 2.3.2. Tribological Characterization

The friction and wear performance of the developed brake composites was evaluated using a chase friction tester (Model XYC-A, Xianyang Xinyi Friction and Sealing Equipment Co., Ltd., Xianyang, China), according to the standard SAE J661-1997 [32]. The test specimen had dimensions of 25.4 mm × 25.4 mm and a flat bottom; the radius of the working surface is conformed to the radius of the test drum. For post-cured brake linings, we removed 1.0/1.2 mm to ensure that the resin-impregnated surface was totally removed. The specimen thickness should be approximately six mm as measured in the center of the specimen. The standard consists of two baseline tests, two fade tests, two recovery tests and a single wear test with the load of 667 N. The interface computer stores the acquired data and displays the test results inline. The abstract of the testing schedule is shown in Table 6.

#### 2.3.3. Morphological Characterization

The worn morphology of the samples after tribology testing was analyzed by SEM (Model SUPRA 55, Carl Zeiss AG, Jena, Germany) and EDS (Model X-Max50, Oxford Instruments Co., Ltd., Oxford, UK), which was used to determine the elements distribution of the worn surface of samples. All samples were coated with a thin layer of gold using a sputtering coater (Model 108, Cressington Scientific Instruments Co., Ltd., Watford, UK).

The surface roughness of the samples after tribology testing was measured by a white light confocal 3D profiler (Model Micromeasure II, Stil Co., Saint Hilaire, France). The measuring range of the area is 3000 μm × 3000 μm, and the step-length of the X and Y axis are both 0.5 μm. Each case was measured three times, after which the results were averaged.

In order to study the properties of the transfer film formed on the counter surface, the tribology tests were carried out by a microcomputer controlled friction test machine (Model MMS-2A, Jinan Yihua Tribology Testing Technology Co., Ltd., Jinan, China). The material of the counter surface is gray iron. The worn morphology of the counterpart after tribological testing was analyzed by SEM (Model S-3400N, Hitachi Co., Ltd., Tokyo, Japan) and EDS (Model QUANTAX, Bruker Nano GmbH, Berlin, Germany), which was used to determine the element distribution of the counter surface.

#### 2.3.4. X-ray Diffraction Analysis

To study the role of La_2_O_3_ in polymeric matrix brake composites during the friction process, the X-ray diffractometer (Model X’pert3 and Empyrean, Panalytical B.V., Almelo, The Netherlands) was used to make a qualitative analysis on the composites. The anode material was Cu. Measurements were obtained with a scanning rate of 0.013°/s and a diffraction angle range increases from 5° to 90° (2-Theta range), where theta is the angle of incidence of the X-ray beam on the sample. The diffraction patterns were analyzed using MDI jade software.

## 3. Results and Discussions

### 3.1. Physical, Mechanical, and Thermo-Physical Properties

The data in Table 7 show that the density and porosity of the brake composites decrease with the decrease in Cu content and increase in La_2_O_3_ content. La_2_O_3_ is less dense than Cu. With the decrease in Cu content, the amount of La_2_O_3_ increases and causes the decrease in density. The porosity of the brake composites decreases in the series because of the closer packing of notably fine La_2_O_3_ powder. The hardness and impact strength of the brake composites increase with the decrease in Cu content and increase in La_2_O_3_ content. Cu is softer than La_2_O_3_. With the decrease in Cu content, the amount of La_2_O_3_ increases and causes the increase in hardness. As mentioned, rare earth oxides can be used in composite materials to improve the mechanical properties [21,22,25,26]. The impact strength, tensile strength, and elastic modulus of the brake composites are increased because of the La_2_O_3_ content as expected, and the results are in accordance with our previous work [25,26]. The thermal performance of the composites occurs in the following order: C_10_L_5_ > C_5_L_10_ > C_15_L_0_ > C_0_L_15_. Composites that contain both Cu and La_2_O_3_ are better than those containing only Cu or La_2_O_3_ in this aspect. We expect that La_2_O_3_ has synergistic effects with Cu in composites, which can further improve the thermal performance of the brake composites.

### 3.2. Tribological Properties

#### 3.2.1. Friction Coefficient

**Figure 3:**
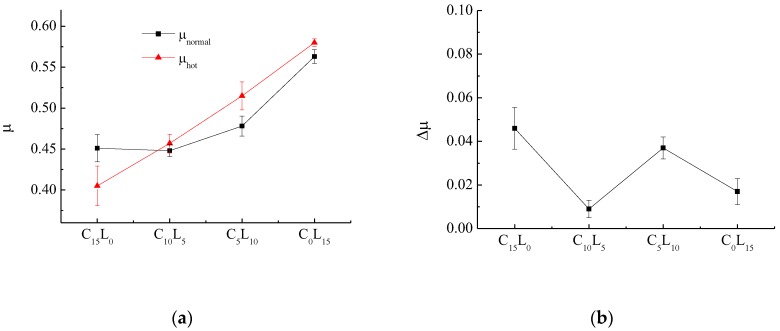
Variation in μ_normal_, μ_hot_ and Δμ (μ fluctuations) with different wt % of Cu and La_2_O_3_ for brake composites: (**a**) μ_normal_ and μ_hot_; (**b**) Δμ.

The variation in friction coefficient (μ) with the decrease in Cu content and increase in La_2_O_3_ content is shown in Figure 3. The normal friction coefficient (μ_normal_) was calculated as the average of selected μ values corresponding to 93, 121, 149, and 204 °C from the second fade cycle. For an ideal automotive brake composites, μ should be 0.20–0.70 (higher value is better). The hot friction coefficient (μ_hot_) was calculated as the average of selected μ values that correspond to 204 and 149 °C of the first recovery cycle, 232, 260, 288, 316, and 343 °C of the second fade cycle, and 260, 204 and 149 °C of the second recovery cycle. Δμ was defined as the difference between μ_normal_ and μ_hot_ to characterize the friction coefficient stability and is shown in Figure 3b.

Figure 3a shows that μ_normal_ and μ_hot_ increase with the decrease in Cu content and increase in La_2_O_3_ content, particularly the increase in μ_hot_. Except for C_15_L_0_, μ_hot_ was higher than μ_normal_ for all composites, which shows the good heating fade resistance of the developed brake composites containing La_2_O_3_. Thus, the addition of La_2_O_3_ effectively improves μ, particularly for the improvement of μ_hot_. In Figure 3b, the La_2_O_3_-containing brake composites has lower Δμ than the La_2_O_3_-free brake composites. C_10_L_5_ has the lowest Δμ, followed by C_0_L_15_ and C_5_L_10_. However, C_15_L_0_ has the highest Δμ, which is not the desired feature. Thus, the addition of La_2_O_3_ is more conducive to the stability of the friction coefficient for brake composites than Cu. C_0_L_15_ has the best friction properties. According to modern tribological theory, sliding friction coefficient, μ, is mainly composed of three kinds of friction coefficients, including μ_a_ caused by adhesion, μ_f_ caused by furrow action and μ_r_ caused by roughness. The sliding friction coefficient, μ, can be given by

μ = μ_a_ + μ_f_ + μ_r_ (3)

where μ_r_ is not related to the composition of the material but is affected by the state of the friction surface. The above results from Table 7 proved that the hardness, tensile strength, and elastic modulus of the brake composites increase with the decrease in Cu content and increase in La_2_O_3_ content. With the increase in tensile strength and elastic modulus of the composites, the shear-resistant of the composite surface is strengthened, leading to the improvement in friction coefficient, which is caused by adhesion (μ_a_). With the increase in the hardness of the composites, the furrow action between the surface of the micro-convex body and its counterpart is strengthened, leading to an improvement in the friction coefficient, which is caused by the furrow action (μ_f_). Therefore, the sliding friction coefficient increases with the increase in La_2_O_3_ content.

#### 3.2.2. Fade and Recovery Behavior

**Figure 4:**
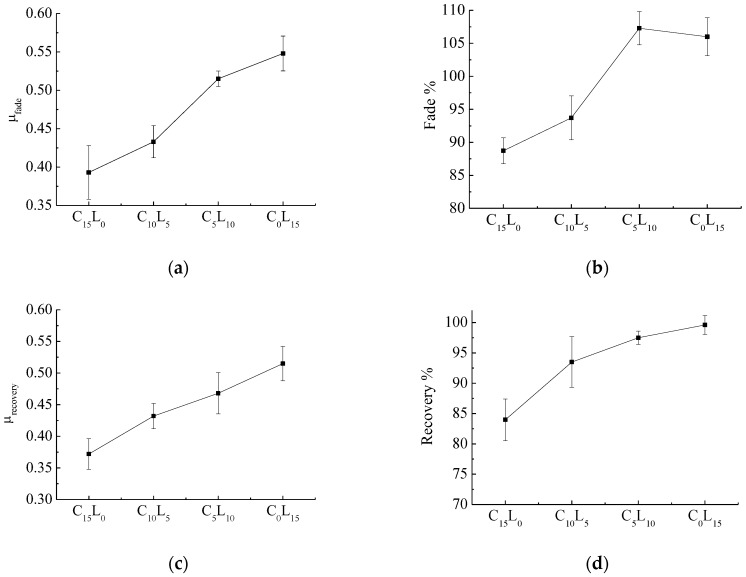
Fade and recovery behaviors of the brake composites: (**a**) μ_fade_; (**b**) fade%; (**c**) μ_recovery_; (**d**) recovery%.

To characterize the fade and recovery properties of all selected composites, various derived friction performance parameters such as μ_fade_, fade% and recovery% were defined as follows: μ_fade_: average μ of the fade and recovery cycles above 260 °C. A higher μ indicates a better heat fading resistance. μ_baseline_: average μ of the first baseline cycle. μ_baseline_ is defined to assess the friction performance without the effect of temperature. fade%: μ_fade_/μ_baseline_ × 100. A higher % fade ratio is desirable. For an ideal brake composites, fade% should be greater than 80%. recovery%: μ_recovery_/μ_baseline_ × 100. A higher recovery% is desirable. In general, for an acceptable brake composites, recovery% should be in the desired range (80%–100%). Figure 4 shows the fade and recovery behaviors of all selected composites.

Figure 4a,b shows that μ_fade_ and fade% almost increase with the decrease in Cu content and increase in La_2_O_3_ content. The added La_2_O_3_ into brake composites has the benefit of improving the heat resistance function. The fade% of C_5_L_10_ and C_0_L_1__5_, whose values are greater than 100, show that μ of the composites dose not decrease with the increase in temperature but increases and proves the good heating fade resistance of the developed brake composites. This tribological property is different from the traditional properties of the brake composites and similar to carbon-carbon composites, which have been widely used in aircraft brake composites because of their excellent tribological properties [33]. It is expected that the addition of La_2_O_3_ in polymeric matrix brake composites will significantly improve the tribological properties. Figure 4c,d shows that μ_recovery_ and recovery% increase with the decrease in Cu content and increase in La_2_O_3_ content. The added La_2_O_3_ into brake composites has the benefit of improving the recovery function. Compared with C_10_L_5_ (composites containing only Cu), C_10_L_5_ (composites containing only La_2_O_3_) show better heat fading resistance and recovery performance. It can be seen that La_2_O_3_ is more conducive to improving the heat fading resistance and recovery performance of the brake composites than copper.

#### 3.2.3. Wear Behavior

**Figure 5:**
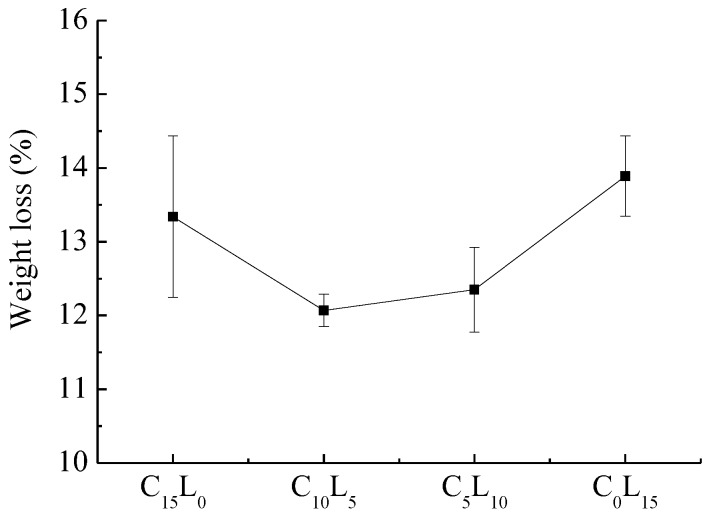
Wear performance of the brake composites.

The wear of the brake composites was measured by the weight loss method after completing various baseline, fade, and recovery cycles. From Figure 5, the weight loss of the composites first decreases and then increases with the decrease in Cu content and increase in La_2_O_3_ content. C_10_L_5_ shows the highest wear resistance, followed by C_5_L_10_. The composite C_15_L_0_ is moderate, whereas C_0_L_15_ is poor in wear performance. Evans and Marshall [34] studied the theory of wear and concluded that the wear loss was directly proportional to the elastic modulus and inversely proportional to the hardness. As mentioned above, with the decrease in Cu content, the amount of La_2_O_3_ increases and causes the increase in hardness and elastic modulus. From Table 7, it can be seen that the hardness firstly increases rapidly at the beginning and then increases slowly with the increase in La_2_O_3_ content, while the elastic modulus increases slowly the first time and then rapidly later. Thus, the wear loss of the composites firstly decreases due to the rapid increase in hardness, and then increases due to the accelerative increase in the elastic modulus. From Figure 5, it also can be seen that the composites that contain both Cu and La_2_O_3_ have better wear performance than those containing only Cu or La_2_O_3_, where similar synergistic effects were observed with the effect on the thermal performance, as shown in Table 7. When the brake composites have poorer thermal conductivity, less heat is conducted away from the friction interface, contributing to the increase in thermal decomposition of the resin and wear loss of the composites.

Thus, overall, C_0_L_15_ is superior to C_15_L_0_ in most properties such as μ_normal_, μ_hot_, Δμ, μ_fade_, μ_recovery_, fade% and recovery%, whereas C_0_L_15_ has slightly superior wear resistance. For brake composites, friction properties are more important than wear resistance. Hence, C_0_L_15_ is the optimal sample in this study for the best possible combination of performance properties.

### 3.3. Worn Surface Analysis

**Figure 6:**
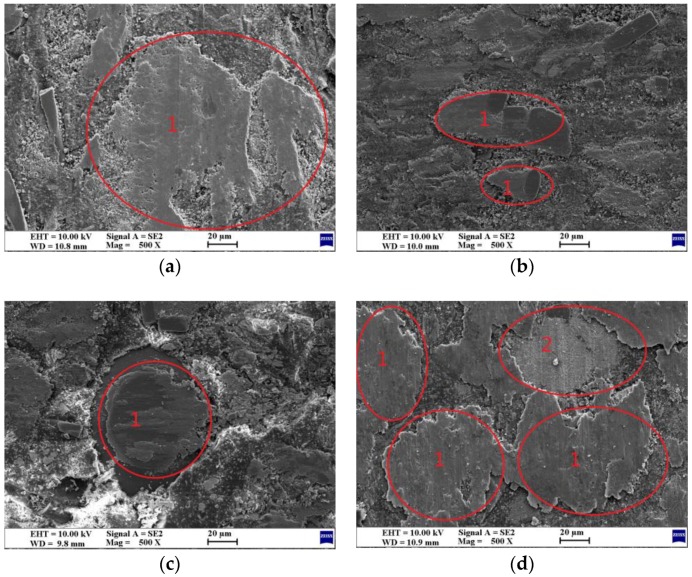
SEM micrographs of the worn surfaces of the brake composites: (**a**) C_10_L_5_; (**b**) C_5_L_10_; (**c**) C_15_L_0_; (**d**) C_0_L_15_.

**Figure 7:**
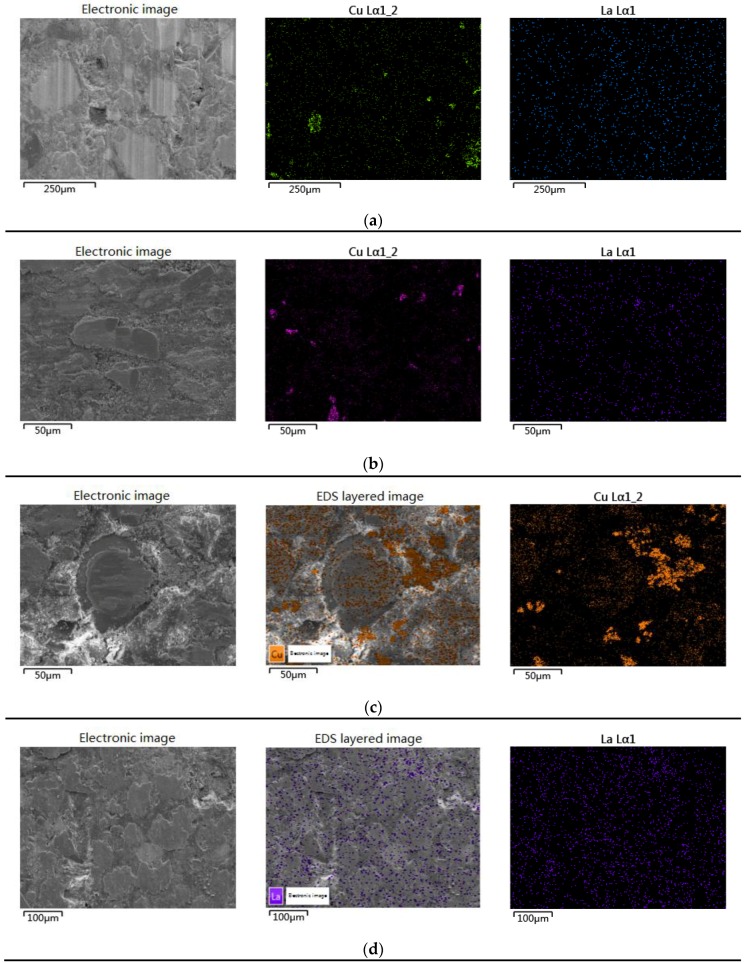
Worn surfaces of composites-EDS elemental mapping images: (**a**) C_10_L_5_; (**b**) C_5_L_10_; (**c**) C_15_L_0_; (**d**) C_0_L_15_.

The wear mechanisms during wearing of friction materials are extremely complex dynamic processes, since several interactions such as physical, chemical, and mechanical are simultaneously operative. Consequently, they depend on various factors such as the basic compositions, their synergistic effect, load, velocity, interface temperature, and friction surface characteristics. It is generally acknowledged by most scholars that a uniform, continuous and thin friction film on the interface is beneficial to the stability of μ and good wear resistance [35,36,37]. The worn surfaces of the brake composites studied by SEM are arranged according to their increasing wear resistance (C_10_L_5_ > C_5_L_10_ > C_15_L_0_ > C_0_L_15_) in Figure 6. Figure 7 shows their EDS micrographs, and the EDS spectrums are given in Figure S1 in the Supplementary Materials.

The distinct features observed in all micrographs in Figure 6 are the thickness and continuity of the friction film (marked as 1), which are possibly enriched with Cu and La_2_O_3_ and must be further confirmed by EDS studies (Figure 7). The micrographs of C_10_L_5_ (Figure 6a) show the smoothest and cleanest surface. A thinner and most continuous film enriched with Cu and La_2_O_3_ (Figure 7a) of uniform thickness was observed throughout the surface with no crack or degradation of ingredients, supporting its highest wear resistance and stable friction behavior. The surface of C_5_L_10_ (Figure 6b) shows discontinuous pieces of friction film enriched with Cu and La_2_O_3_ (Figure 7b), which was responsible for its higher wear resistance. The micrographs of C_15_L_0_ (Figure 6c) show some damage to the ingredients, wear debris, and the thinnest but discontinuous film enriched with Cu (Figure 7c), which was responsible for some wear. The surface of C_0_L_15_ (Figure 6d) shows the thickest friction film enriched with La_2_O_3_ (Figure 7d). A small amount of thick friction film (marked as 2) was easily abraded from the surface, which caused more wear.

By contrast with the EDS elemental mapping images of the worn surface of C_15_L_0_ (Figure 7c) and C_0_L_15_ (Figure 7d), lanthanum oxide is more uniformly distributed on the surface than copper, which is more conducive to the formation of a continuous friction film. The lanthanum oxides are allotropes of hexagonal crystals, which are similar to layered structures, possessing high melting points and a low hardness. From Figure 7d, it can be seen that the friction film enriched with La coated on the worn surface, which can act as a solid lubricant at high temperatures as expected [24]. With more La_2_O_3_, a thicker friction film is formed. However, the thick oxide film is more easily worn out from the surface as C_0_L_15_, which is not desirable.

### 3.4. Three-Dimensional Surface Texture Analysis

**Figure 8:**
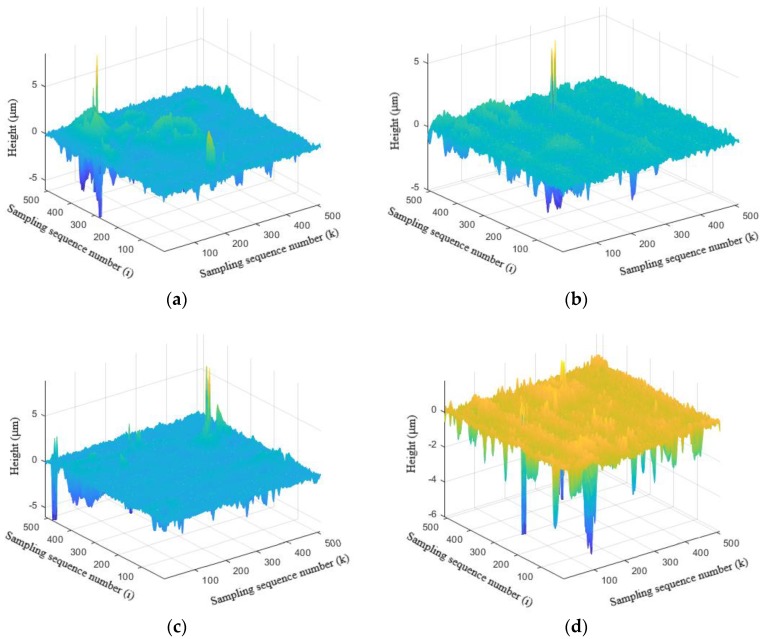
3-D surface topography of the worn surfaces of the brake composites: (**a**) C_15_L_0_; (**b**) C_10_L_5_; (**c**) C_5_L_10_; (**d**) C_0_L_15_.

As previously mentioned, the friction coefficient caused by roughness, μ_r_, is affected by the state of the friction surface. The measurements of 3-D surface topography of the worn surfaces of the brake composites after tribology testing are shown in Figure 8. The obtained roughness parameters of the worn surface roughness of specimens are shown in Table 8.

*S_a_* shows the deviation of surface height, and can be expressed as

(4) $$S_{a}=\frac{1}{MN}\sum_{i=0}^{M-1}\sum_{k=0}^{N-1}\left|z(x_{i},y_{k})-u\right|$$

where *z(x_k_, y_l_)* is the height of the Z axis at the coordinate point, *M* is the length of 3-D surface image, *N* is the width of 3-D surface image, and *u* can be computed by

(5) $$u=\frac{1}{MN}\sum_{i=0}^{M-1}\sum_{k=0}^{N-1}z(x_{i},y_{k})$$

*S_q_* shows the standard deviation of surface height, and can be given by

(6) $$S_{q}=\sqrt{\frac{1}{MN}\sum_{i=0}^{M-1}\sum_{k=0}^{N-1}{\left[z(x_{i},y_{k})-u\right]}^{2}}$$

*S_ku_* shows the steepness of surface topography, and can be defined as

(7) $$S_{ku}=\frac{1}{MNS_{q}{}^{4}}{\sum_{i=0}^{M-1}\sum_{k=0}^{N-1}\left[z(x_{i},y_{k})-u\right]}^{4}$$

where *S_ku_* = 3, *S_ku_* < 3, and *S_ku_* > 3 are interpreted as a normal distribution, the probability dispersion of height distribution, and the probability concentration of height distribution, respectively. The smaller S_ku_ is, the more amounts of surface peaks will be.

From Figure 8 and Table 8, it can be seen that the value of *S_q_* of the brake composites containing La_2_O_3_ is much lower than that containing Cu under the same condition, indicating that the brake composites containing La_2_O_3_ have a lower surface roughness during the friction process. As mentioned above, La_2_O_3_ is more conducive to the formation of friction film than Cu and causes the decrease in surface roughness. The values of *S_ku_* in Table 8 are all greater than three, indicating that all the specimens surface have surface peaks. With the decrease in Cu content, the amount of La_2_O_3_ increases and causes the increase in the values of *S_ku_*. It can be seen that the surface of C_0_L_15_ demonstrates more peaks than any other types. As the amount of peaks increases on the surface, the furrow action enhances and causes the increase in friction coefficient. Thus, C_0_L_15_ has the best friction properties.

### 3.5. Friction Transfer Film

**Figure 9:**
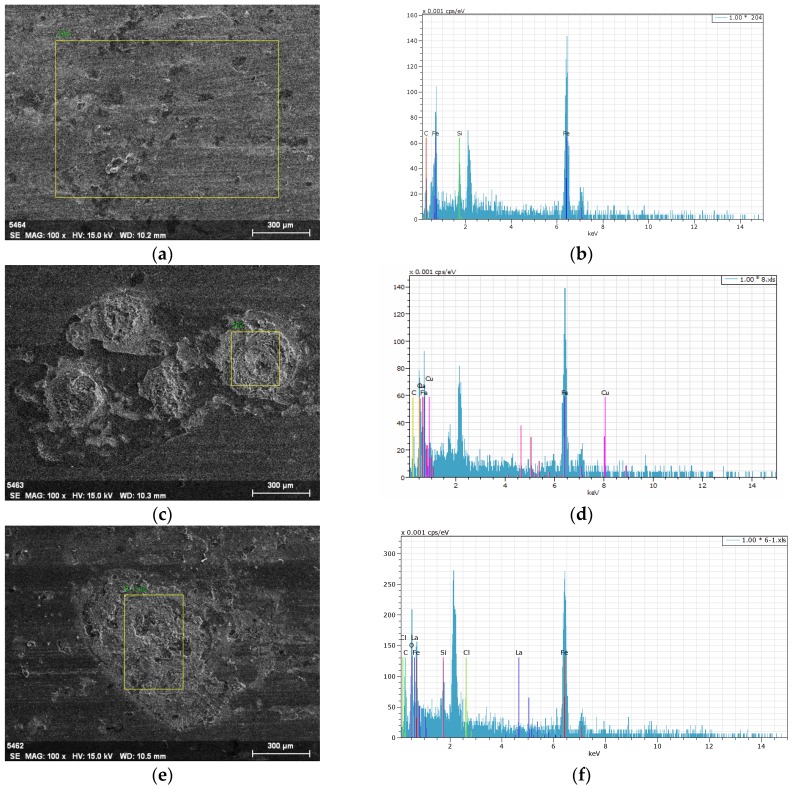
SEM micrographs and EDS spectrums of the counter surface: (**a**) SEM micrograph of the counter surface before tribology testing; (**b**) EDS spectrum of the counter surface before tribology testing; (**c**) SEM micrograph of the counter surface after tribology testing with C_15_L_0_; (**d**) EDS spectrum of the counter surface after tribology testing with C_15_L_0_; (**e**) SEM micrograph of the counter surface after tribology testing with C_0_L_15_; (**f**) EDS spectrum of the counter surface after tribology testing with C_0_L_15_.

The tribological properties of the brake composites is directly affected by the thickness, uniformity, and continuity of the transfer film formed on the counter surface. Many research results indicated that copper has a good ductility, which is conducive to the formation of stable, continuous and uniform friction transfer film on the counter surface, and plays a role in stabilizing the friction coefficient and reducing wear loss [9,10,11,38]. In order to study whether La_2_O_3_ could play a role similar to Cu during the friction process, the coupled parts before and after tribology testing with C_15_L_0_ and C_0_L_15_ were analyzed by SEM and EDS. The SEM micrographs and EDS spectrums of the counter surface are shown in Figure 9. From Figure 9, compared with the coupled part before tribology testing (Figure 9a), transfer films were formed on the counter surface after tribology testing (Figure 9c,e), which were further confirmed as films enriched with Cu and La_2_O_3_ (Figure 9d,f), respectively. Compared with Figure 9c, the transfer layer formed on the counter surface after tribology testing with C_0_L_15_ (Figure 9e) was more continuous and compacted, helping to the better tribological properties of C_0_L_15_ than C_15_L_0_. It can be seen that the addition of La_2_O_3_ to brake composites is more conducive to the formation of uniform and continuous transfer film on the counter surface than Cu. Overall, rare earth La_2_O_3_ can be successfully used as a replacement for Cu in brake composites.

### 3.6. X-ray Diffraction Analysis

**Figure 10:**
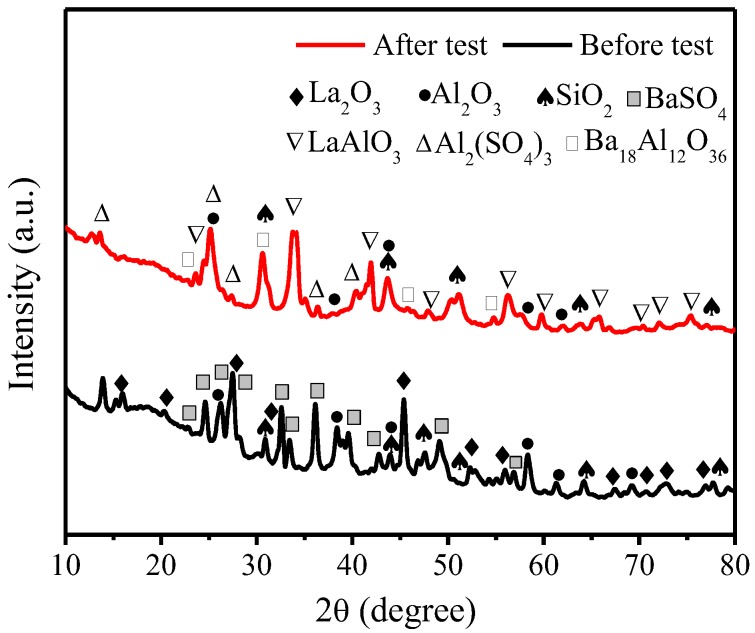
XRD patterns of C_0_L_15_ before and after tribology testing.

To further study the role of La_2_O_3_ during friction and wear processes, the qualitative phase analysis of C_0_L_15_ before and after tribology testing was carried out with X-ray diffraction (XRD). The XRD patterns are shown in Figure 10. It can be seen that C_0_L_15_ before tribology testing appears to have diffraction peaks of La_2_O_3_, Al_2_O_3_, SiO_2_ and BaSO_4_, which are consistent with the composition of C_0_L_15_. Compared with C_0_L_15_ before tribology testing, C_0_L_15_ after tribology testing appears to have diffraction peaks of Al_2_O_3_ and SiO_2_, and the new diffraction peaks of LaAlO_3_, Ba_18_Al_12_O_36_ and Al_2_(SO_4_)_3_, while the diffraction peaks for the La_2_O_3_ and BaSO_4_ phase disappear, suggesting that the chemical reaction occurs during the friction and wear processes.

The friction and wear processes of the brake composites are extremely complex dynamic processes, since several interactions such as physical, chemical, and mechanical are simultaneously operative. Under the interaction of external force, chemistry, heat and rare earth lanthanum oxide with excellent chemical activity, the chemical reaction occurs among components. The reaction between La_2_O_3_ and Al_2_O_3_ can form LaAlO_3_. The chemical equation can be expressed as

(8) $${\mathrm{La}}_{2}O_{3}+2{\mathrm{Al}}_{2}O_{3}\overset{\Delta }{\to }{2\mathrm{LaAlO}}_{3}$$

BaSO_4_ reacting with Al_2_O_3_ yields Ba_18_Al_12_O_36_ and Al_2_(SO_4_) _3_. The chemical equation can be given by

(9) $${18\mathrm{BaSO}}_{4}+12{\mathrm{Al}}_{2}O_{3}\overset{\Delta }{\to }{\mathrm{Ba}}_{18}{\mathrm{Al}}_{12}O_{36}+6{\mathrm{Al}}_{2}{{(\mathrm{SO}}_{4})}_{3}$$

Compared with La_2_O_3_, LaAlO_3_ shows hexagonal crystals as well [39], which is similar to layered structures with lower melting points and lower hardness, and is more conducive to the formation of continuous and compacted friction films. LaAlO_3_ can be used in functional ceramics with high quality [40]. The generated Ba_18_Al_12_O_36_ belongs to the barium aluminate salts, which can be used as inorganic bonding materials, and has excellent high temperature properties [41]. The produced Al_2_SO_4_ has a melting point of 770 °C, and can be used as an inorganic binder [42]. Given the above, the addition of La_2_O_3_ to the brake composites can lead to the reaction between La_2_O_3_ and Al_2_O_3_ to form LaAlO_3_, and lead to the reaction between Al_2_O_3_ and BaSO_4_ to produce Ba_18_Al_12_O_36_ and Al_2_SO_4_ during the friction and wear processes. This process is similar to the mechanism of sintering ceramics that enables the previously accumulated inorganic filler to bind together with chemical bonds, which can effectively improve the tribological properties of the brake composites at elevated temperature. The traditional resin-based brake materials will suffer from the decrease of friction coefficient at elevated temperature, owing to the limited heat resistance of the phenolic resin. As previously mentioned, μ_hot_ for all developed brake composites containing La_2_O_3_ was higher than that of μ_normal_. This tribological property of the composites is opposite to the traditional resin-based brake composites, and is in accordance with the property of the ceramic brake composites at elevated temperature [43,44]. Overall, the addition of La_2_O_3_ to the brake composites can promote chemical reaction between inorganic fillers, and act as an inorganic binder at elevated temperature, which can effectively improve the tribological properties of the brake composites.

## 4. Conclusions

This study aims to use its findings as a guide to the development of Cu -free, metal-free and eco-friendly brake composites. The results obtained are useful for understanding the mechanism of the role of La_2_O_3_ in polymeric matrix brake composites to replace Cu. Cu can be successfully replaced by La_2_O_3_ in brake composites. The results are summarized as follows:

1. For almost all important properties (e.g., μ_normal_, μ_hot_, Δμ, μ_fade_, μ_recovery_, fade% and recovery%), Cu-free brake composites (C_0_L_15_) performed significantly better than the brake composites containing Cu without La_2_O_3_ (C_15_L_0_), while the Cu-contained brake composites (C_15_L_0_) are slightly superior in thermal performance and wear resistance.
2. La_2_O_3_ has synergistic effects with Cu in the composites, which can further improve the thermal performance and wear resistance of the brake composites. Composites containing both Cu and La_2_O_3_ have better thermal and wear performance than those containing only Cu or La_2_O_3_.
3. The addition of La_2_O_3_ in brake composites improves the heat resistance function compared to the addition of Cu. The friction coefficient (μ) increases with the increase in temperature, which is different from the traditional properties of the brake composites and proves the best heating fade resistance of the developed copper -free brake composites.
4. Compared with the addition of Cu in brake composites, La_2_O_3_ is more conducive to the formation of continuous and compacted friction films and transfer films and causes the decrease in surface roughness, which is beneficial to the tribological properties of the brake composites.
5. The addition of La_2_O_3_ to the brake composites can cause the reaction between La_2_O_3_ and Al_2_O_3_ to form LaAlO_3_, and cause the reaction between Al_2_O_3_ and BaSO_4_ to produce Ba_18_Al_12_O_36_ and Al_2_SO_4_ during the friction and wear processes. This process is similar to the mechanism of sintering ceramics, which can effectively improve the tribological properties of the brake composites at elevated temperature.

## Figures and Tables

**Table 1:** Details of La_2_O_3_ used in brake composites.

Property Details	Purity (%)	Granularity (mesh)	Melting Point (°C)	Density (g·cm^−3^)
Specifications	99.95	200	2217	6.51

**Table 2:** Details of the ceramic fibers used in brake composites.

Property Details	Fiber Length (mm)	Fiber Diameter (μm)	Mohs Hardness	Refractoriness (°C)	Main Chemical Components (%)
Specifications	1.0–3.5	2.0–4.0	5–6	>1170	SiO_2_: 45–55 Al_2_O_3_: 40–50

**Table 3:** Specifications of the resin used in brake composites.

Sieve Analysis Test 160-mesh Sieve (%)	Curing Time 150 °C (s)	Flow Distance 125 °C (mm)	Viscosity (mPa·s)	Relative Molecular Mass	pH	Free Phenol (%)
≤5	50–100	40–80	3–4	600–700	>7	≤5

**Table 4:** Details of Cu used in brake composites.

Property Details	Purity (%)	Granularity (mesh)	Melting Point (°C)	Density (g·cm^−3^)
Specifications	99.5	200	1083.4	8.96

**Table 5:** Formulation design and designations of composites.

Ingredients/Designation	Cu (wt %)	La_2_O_3_ (wt %)	Parent Composition ^1^ (wt %)
C_15_L_0_	15	0	85
C_10_L_5_	10	5	
C_5_L_10_	5	10	
C_0_L_15_	0	15	

^1^ Binder (phenolic resin)–20 wt %; fibres (ceramic)–15 wt %; additives (graphite)–3 wt % and fillers (alumina, barite, NBR powder)–47 wt %.

**Table 6:** Chase testing schedule.

S. no.	Test Runs	Speed (rpm)	Load (N)	On Time (s)	Off Time (s)	Repetitions	Temperature Range (°C)
1	First Baseline	417	667	10	20	20	82–93
2	First fade	417	667	600	0	1	82–288
3	First recovery	417	667	10	0	1	260–93
4	Wear	417	667	20	10	100	193–216
5	Second fade	417	667	600	0	1	93–343
6	Second recovery	417	667	10	0	1	316–93
7	Second Baseline	417	667	10	20	20	82–93

**Table 7:** Properties of the developed composites.

Properties	C_15_L_0_		C_10_L_5_		C_5_L_10_		C_0_L_15_	
	Average	Standard Deviation	Average	Standard Deviation	Average	Standard Deviation	Average	Standard Deviation
Density (g·cm^−3^)	2.24	0.01	2.22	0.02	2.15	0.03	2.09	0.02
Porosity (%)	0.57	0.03	0.51	0.04	0.42	0.02	0.40	0.01
Hardness (HRM)	103.2	0.6	109.1	0.5	111.9	1.0	112.9	0.4
Impact strength (kJ/m^2^)	7.13	0.23	7.54	0.09	8.13	0.34	9.90	0.20
Tensile strength (MPa)	37.94	0.51	38.96	0.57	39.20	0.39	41.24	0.43
Elastic modulus (Gpa)	1.30	0.04	1.37	0.03	1.43	0.06	1.58	0.07
Thermal conductivity (W/m·K)	1.83	0.04	1.95	0.05	1.88	0.02	1.79	0.03
Thermal resistance (×10^−4^ K·m^2^/W)	16.70	0.37	15.40	0.41	16.04	0.17	17.30	0.29

**Table 8:** Roughness parameter of the worn surface roughness of specimens.

Roughness Parameters	C_15_L_0_		C_10_L_5_		C_5_L_10_		C_0_L_15_	
	Average	Standard Deviation	Average	Standard Deviation	Average	Standard Deviation	Average	Standard Deviation
*S_a_* (μm)	0.44	0.02	0.22	0.01	0.23	0.01	0.21	0.01
*S_q_* (μm)	0.76	0.03	0.36	0.01	0.42	0.01	0.41	0.01
*S_ku_*	12.90	1.35	28.48	1.85	46.26	1.59	55.02	1.87

## Supplementary Materials

The following are available online at http://www.mdpi.com/2073-4360/10/9/1027/s1, Figure S1: EDS spectrums of the worn surfaces of the brake composites: (a) C_10_L_5_; (b) C_5_L_10_; (c) C_15_L_0_; (d) C_0_L_15_.
