# Supplementary material for: The Role of Rare Earth Lanthanum Oxide in Polymeric Matrix Brake Composites to Replace Copper
Source: Polymers (Basel). 2018 Sep 14;10(9):1027. doi: 10.3390/polym10091027 (PMC6403813; doi:10.3390/polym10091027)
Supplement: Supplementary file 1 [file polymers-10-01027-s001.pdf]

polymers

MDPI

Supplementary

The role of rare earth lanthanum oxide in polymeric matrix brake composites to replace copper

Kaikui Zheng [1,2], Chenghui Gao [1,*], Fushan He [1] and Youxi Lin [1]

[1] School of Mechanical Engineering and Automation, Fuzhou University, Fuzhou 350116, China; kuikui@fzu.edu.cn (K.Z.); hfshan@fzu.edu.cn (F.H.); lyx@fzu.edu.cn (Y.L.)
[2] Mechanical and Electrical Engineering Practice Center, Fuzhou University, Fuzhou 350116, China
* Correspondence: gch@fzu.edu.cn; Tel.: +86-0591-2286-6810

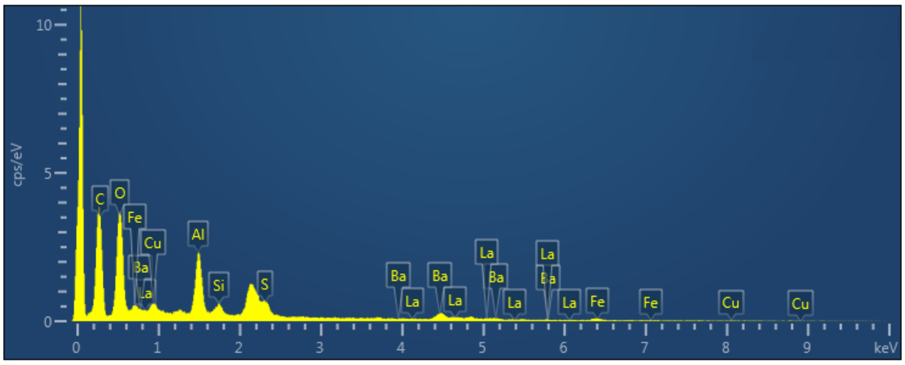

(**a**)

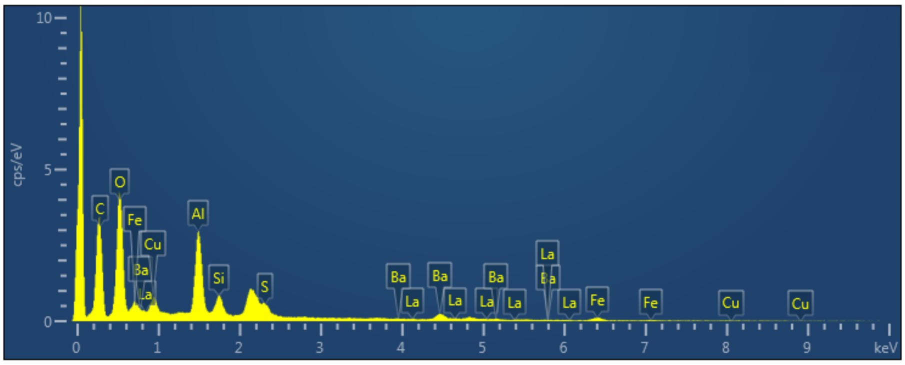

(**b**)

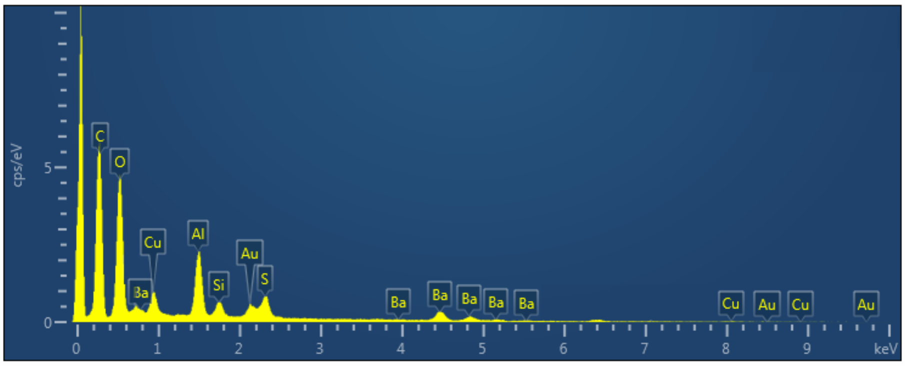

(**c**)

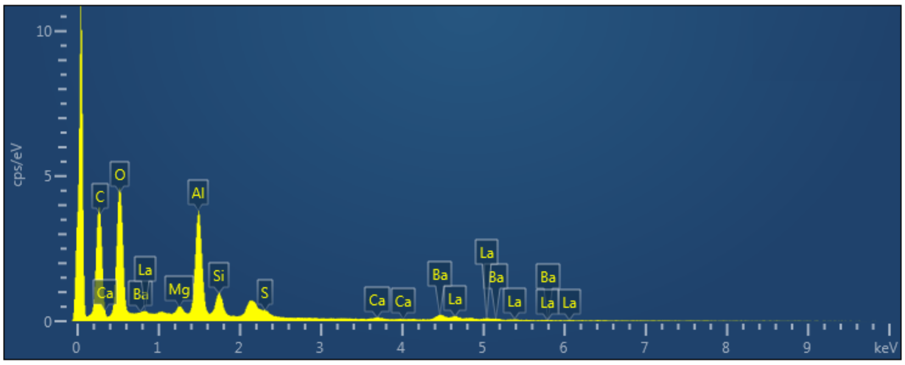

(**d**)

Figure 1. EDS spectrums of the worn surfaces of the brake composites: (**a**) $C_{10}L_5$; (**b**) C_5L_{10}; (**c**) $C_{15}L_0$; (**d**) C_0L_{15}.

© 2018 by the authors. Submitted for possible open access publication under the terms and conditions of the Creative Commons Attribution (CC BY) license (http://creativecommons.org/licenses/by/4.0/).
